# Concavity compression, liner constraint, and instability risk in cuff-deficient 135° reverse shoulder arthroplasty

**DOI:** 10.1016/j.jsea.2026.100046

**Published:** 2026-06-05

**Authors:** Stefan Bauer, Igor Shirinskiy, William G. Blakeney

**Affiliations:** Centre de Chirurgie de l'Épaule, du Coude et de la Main, Ensemble Hospitalier de la Côte (EHC), Morges, Switzerland; UWA Medical School, Division of Surgery, University of Western Australia, Perth, Australia; Centre de Chirurgie de l'Épaule, du Coude et de la Main, Ensemble Hospitalier de la Côte (EHC), Morges, Switzerland; Department of Human Movement Sciences, Vrije Universiteit Amsterdam, Amsterdam, The Netherlands; UWA Medical School, Division of Surgery, University of Western Australia, Perth, Australia; Royal Perth Hospital, Perth, Australia

Dear Editors,

We read with great interest the article by Boubekri et al^3^ entitled “Early radiographic findings are not associated with instability for reverse total shoulder arthroplasty with the Perform metaphyseal-fixated humeral stem” in the *Journal of Shoulder and Elbow Arthroplasty*.

The authors should be congratulated for providing important clinical and radiographic data from a highly experienced surgeon of a new 135° reverse shoulder arthroplasty (RSA) system.

However, we believe the presented data may support an additional and clinically important interpretation.

Pioneering work by Lippitt and Matsen^5^^,^^6^ demonstrated the biomechanical principle that may help explain increased instability observed in cuff-deficient 135° RSA.

“The greater the arc provided by the glenoid, the larger the range of joint force angles acting through the humeral head that may be stabilized.”

Lippitt and Matsen further demonstrated that glenohumeral stability is direction-dependent and governed by the interaction between glenoid concavity and joint reaction force orientation. In cuff-deficient shoulders, lowering the neck-shaft angle (NSA) may shift the resultant joint reaction force toward a superior-lateral shear-dominant direction relative to the available prosthetic concavity, thereby reducing effective concavity-compression, particularly when combined with reduced liner constraint.

The Frankle classification^1^ underlines the following 3 central biomechanical mechanisms:

(1) Altered concavity-compression,
(2) Reduced concavity-containment,
(3) Impingement acting as a translational displacing force.

Boubekri et al’s manuscript postulates that there is no association between varus alignment and instability. However, the study is insufficiently powered to definitively exclude such an association. The cited study by Bauer et al^2^ did not propose stem malalignment as the sole mechanism of instability. Rather, instability was interpreted as a multifactorial phenomenon involving cuff deficiency, reduced liner stability, and low-NSA configuration, with varus alignment representing only 1 factor observed in a single instability case.

Notably, all 8 instability events in Boubekri et al’s study occurred exclusively with massive rotator cuff tears or cuff tear arthropathy (CTA), corresponding to an instability rate of 12.1% (8/66) within this cuff-deficient subgroup of 66 shoulders. Additionally, although the stem itself has a 135° NSA, 91.3% of shoulders were treated with a 10° angled liner, substantially altering containment geometry and effective directional constraint. This distinction is relevant when comparing instability behavior across 135° RSA systems.

In the same subgroup, Bauer et al’s instability rate was 7% (4/57) although they did not use 10° liners like Boubekri et al. The associated introduction of the two-hand lever test^2^ may partly explain the reduced instability rate despite the use of a potentially less stable liner.

In Boubekri et al’s series, 7 of the 8 instability cases occurred with use of the standard 0.45 capture ratio (CR) liner. However, the exact number of cuff-deficient shoulders treated with the 0.45 liner is not reported. Consequently, the instability rate of this high-risk subgroup cannot be calculated precisely from the published data.

Nevertheless, the available data allow estimation of a clinically important instability spectrum. Since 7 instability events occurred in cuff-deficient shoulders treated with a 0.45 liner, the instability rate of this subgroup would range from:

• 10.8% (7/65) if all 65 potentially eligible cuff-deficient shoulders received a 0.45 liner, to
• 21.2% (7/33) if approximately half (33 shoulders) received a 0.45 liner.

This suggests that the combination of cuff deficiency and reduced liner constraint may represent a high-risk biomechanical environment that may not be adequately captured by analysis of the overall cohort alone.

This observation is noteworthy since RSA was originally developed for CTA.^4^

CR alone may not adequately characterize biomechanical prosthetic stability. In contrast, liner stability ratio^7^ and the emerging inclination-adjusted liner stability ratio, both requiring further validation, provide a more comprehensive biomechanical quantification of prosthetic stability, integrating the influence of liner orientation, and force-vector direction. Consequently, constructs with identical nominal CR may exhibit substantially different stability.

Interestingly, the authors themselves acknowledge this potential signal, noting that the senior surgeon subsequently transitioned away from the 0.45 standard liner in favor of the more retentive 0.55 liner. A new 0.65 liner has since been launched. This clinical evolution further supports the concept that increasing liner constraint may improve stability with a 135° NSA.

We therefore believe the present study may provide additional indirect clinical support for the importance of concavity compression, vector direction, and liner stability in 135° RSA, particularly in cuff-deficient patients.

An important study by Pandy et al^8^ also found that glenoid shear forces were broadly similar across native and RSA configurations, whereas compressive loading varied with implant design and cuff contribution. This observation supports a more nuanced interpretation: instability risk likely depends less on a single radiographic or implant parameter and more on whether the construct preserves sufficient compression and containment in the direction of the applied joint reaction force.

We would appreciate a detailed reply from the authors regarding the exact breakdown of instability events across the relevant subgroups. Specifically, it would be informative to know the exact instability rate among the 65 shoulders (massive rotator cuff tear + CTA) treated with the 0.45 liner, as this subgroup appears to represent the clinically most relevant high-risk population.

Reporting this denominator would help determine whether the observed instability signal reflects an interaction between cuff deficiency and reduced prosthetic constraint, rather than being inadequately captured by analysis of the overall cohort alone.

We congratulate the authors for their contribution to this evolving field.

Sincerely,

Stefan Bauer, MD, Igor Shirinskiy, MD, and William G. Blakeney, MD.

## Disclaimers:

Funding: No funding was disclosed by the authors.

Conflicts of interest: The author, their immediate family, and any research foundation with which they are affiliated have not received any financial payments or other benefits from any commercial entity related to the subject of this article.
